# Merkel Cell Polyomavirus in colorectal cancer: exploring the Wnt/β-catenin pathway

**DOI:** 10.1016/j.virusres.2026.199809

**Published:** 2026-09-24

**Authors:** Sara Passerini, Maria Dolci, Sara Messina, Valentina Alyssa Caterina, Lucia Signorini, Francesca Camurri, Marco Graziani, Giorgia Gallo, Giovanni Di Nardo, Serena Delbue, Valeria Pietropaolo

**Affiliations:** a Department of Public Health and Infectious Diseases, Sapienza University of Rome, Rome, Italy; b Department of Biomedical, Surgical and Dental Sciences, University of Milan, Milan, Italy; c Pediatric Gastroenterology and Endoscopy Unit, Department of Pediatric Specialties, Santobono Pausilipon Children's Hospital, Naples, Italy; d Department of Neurosciences, Mental Health and Sensory Organs (NESMOS), Sapienza University of Rome, Rome, Italy

**Keywords:** Merkel cell polyomavirus, Colorectal cancer, Wnt/β-catenin pathway, Tumorigenesis

## Abstract

Merkel cell polyomavirus (MCPyV) is the established oncogenic driver of Merkel cell carcinoma (MCC), but its role in other malignancies is under investigation. Although MCPyV DNA has been detected in colorectal cancer (CRC) specimens, its biological relevance and contribution to colorectal tumorigenesis remain unclear. In this observational study, we investigated MCPyV prevalence, load and molecular status in 122 CRC specimens and peritumoral tissues. In addition, to extend beyond DNA detection, transcriptional profiling was performed to assess early (*LTAg*) and late (*VP1*) transcripts, and viral microRNAs. Furthermore, the mRNA expression levels of key Wnt signaling components, including *CTNNB1, MYC* and *CCND1* were examined. *AXIN2* was also used as a surrogate marker of pathway activation. Our findings revealed similar MCPyV prevalence and load between tumor and adjacent healthy tissues, suggesting a wide colonization of the colorectal mucosa. Sequencing analyses showed canonical NCCR and VP1 structures, and full length LTAg, with no evidence of viral integration. These data, combined with the detection of both early and late transcripts, indicate an episomal and transcriptionally active state in colorectal tissues. Notably, virus-positive tissues exhibited an over-expression of *CTNNB1*and its target genes across all clinical subgroups, regardless of anatomical site or tumor stage. Our results are congruent with the establishment of persistent MCPyV infection in the colorectal mucosa, potentially characterized by transcriptionally active episomal genomes. Furthermore, upregulation of the Wnt/β-catenin cascade in virus-positive tissues suggests that MCPyV may exploit this host pathway to drive cellular proliferation. A limitation of this study is lack of comparison to colorectal mucosa in patients without CRC. Overall, these findings are consistent with widespread viral colonization of the colorectal mucosa in a subset of CRC patients, and raise the hypothesis that MCPyV may modulate host signaling pathways relevant to colorectal biology. Whether MCPyV plays any etiological role in colorectal carcinogenesis, or whether its detection is coincidental, cannot be determined from the present observational data.

## Highlights

• Merkel Cell Polyomavirus (MCPyV) prevalence and viral load were similar between colorectal tumors and peritumoral tissues, suggesting a wide colonization of the colorectal mucosa.
• The virus establishes persistent infection in colorectal tissues, characterized by episomal and transcriptionally active genomes.
• MCPyV-positive tissues showed significant over-expression of *β-catenin* and its target genes, *c-myc* and *cyclin D1*.
• Activation of the Wnt/β-catenin cascade suggests that MCPyV may contribute as a cofactor to colorectal tumorigenesis.

## Graphical abstract

**Figure:**
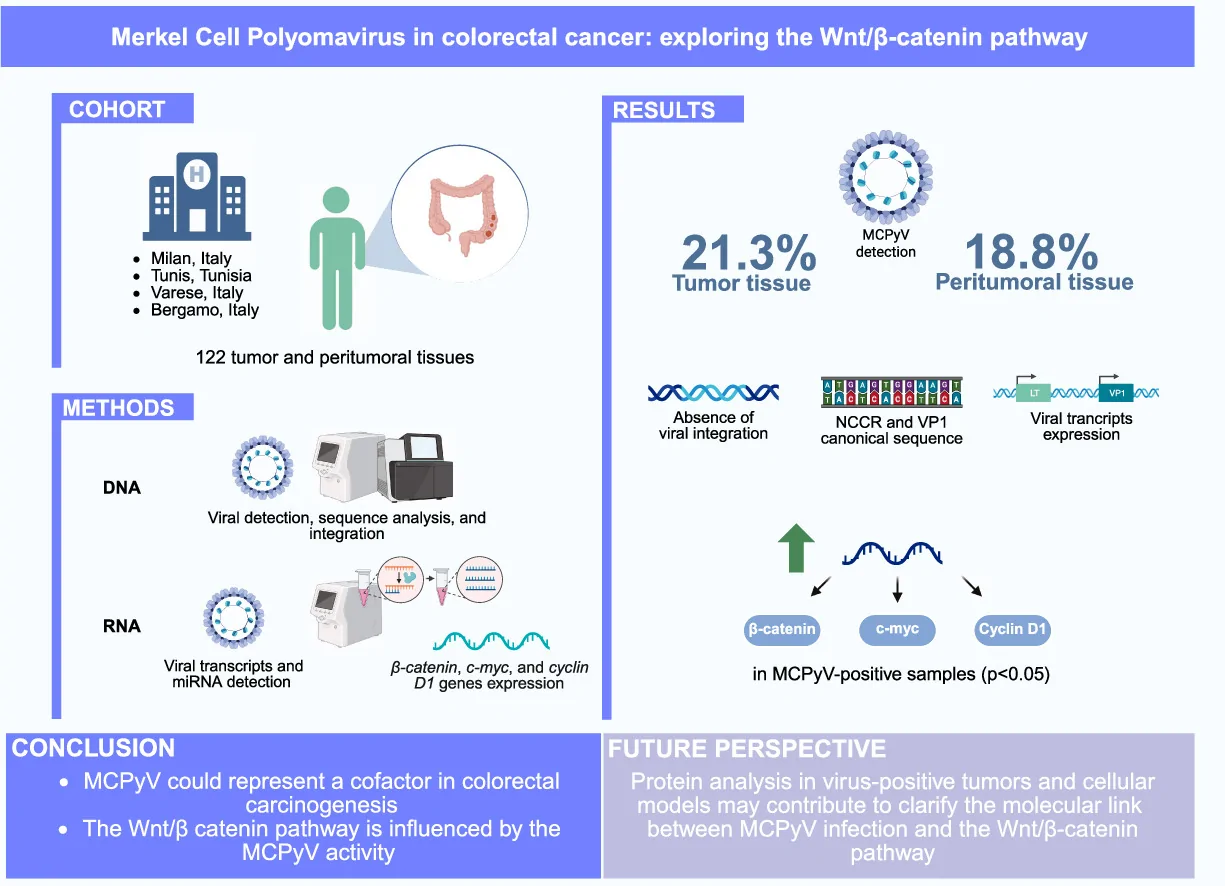

## Abbreviation

CRC: Colorectal cancer
CD: Crohn’s disease
UC: Ulcerative colitis
IBDs: Inflammatory bowel diseases
EBV: Epstein-Barr virus
HPV: Human papillomavirus
HCMV: Human cytomegalovirus
HCV: Hepatitis C virus
HPyVs: Human polyomaviruses
LTAg: Large T antigen
APC: Adenomatous polyposis coli
JCPyV: JC Polyomavirus
SV40: Simian Virus 40
BKPyV: BK Polyomavirus
MCPyV: Merkel Cell Polyomavirus
sTAg: small T antigen
ALTO: alternative LT open reading frame
VP: Viral Protein
microRNA: miRNA
NCCR: non-coding control region
MCC: Merkel cell carcinoma
Rb: Retinoblastoma
qPCR: quantitative polymerase chain reaction
DIPS: detection of the integrated papilloma sequence
RT: reverse-transcribed
IQR: interquartile range
LMP1: Latent Membrane Protein 1
PP2A: protein phosphatase 2A

## 1 Introduction

Colorectal cancer (CRC) is a highly prevalent malignancy worldwide, representing the third most commonly diagnosed cancer, characterized by a high mortality rate in both males and females. (Siegel et al., 2023) Colorectal carcinogenesis is a complex, multistep process driven by genetic predisposition, dietary habits, host-microbiota interactions, and chronic inflammatory stimuli. (O'Keefe, 2016; Bachir et al., 2026) Among them, chronic intestinal inflammation is a well-established risk factor for CRC. Notably, patients with Crohn’s disease (CD) and ulcerative colitis (UC), collectively referred to as inflammatory bowel diseases (IBDs), face an increased risk of developing CRC compared to the general population. (Fanizza et al., 2024) In addition, microbial infections have been increasingly implicated as contributors to sporadic CRC development. (Lucas et al., 2017) In this framework, microbial species, modulated by nutritional and environmental factors, act synergistically with the host inflammatory microenvironment to drive CRC initiation, progression, and metastasis. Underscoring these dynamics, recent metagenomic and transcriptomic studies have identified distinct bacterial, fungal, and viral signatures enriched in CRC patients compared to healthy controls, highlighting the functional role of host-microbiota interactions in colorectal pathogenesis. (Emlet et al., 2020; Qian et al., 2025;; Torshizi Esfahani et al., 2025; Zhang et al., 2026) Among the infectious agents identified within this microenvironment, viruses have gained increasing attention, although their definitive contribution to CRC pathogenesis remains controversial and incompletely understood. (Marongiu and Allgayer, 2022) Several viruses, including Epstein-Barr virus (EBV), Human papillomavirus (HPV), Human cytomegalovirus (HCMV), and Hepatitis C virus (HCV), have been evaluated for their plausible role in CRC. (Bai et al., 2016; Jafari Maskouni et al., 2023; Chen et al., 2025; Yang et al., 2026) Moreover, Human polyomaviruses (HPyVs) have been proposed as candidate viral contributors to CRC due to their lifelong persistence in the host and to the expression of transforming proteins, particularly the Large T antigen (LTAg). (Prado et al., 2018; Marongiu and Allgayer, 2022) Mechanistically, viral infections are hypothesized to promote tumor development through multiple pathways, including the induction of chronic inflammation, immune evasion, genomic instability, and the direct modulation of cellular signaling, either via direct infection of epithelial cells or through interactions within the tumor microenvironment. (Xiao et al., 2025)

A primary target of this viral modulation is the aberrant activation of the Wnt/β-catenin pathway, a well-established hallmark of CRC initiation and progression. (Li et al., 2024) Constitutive activation of this pathway, frequently resulting from alterations in the adenomatous polyposis coli (APC) gene, promotes nuclear accumulation of β-catenin and transcriptional activation of target genes such as *MYC* and *CCND1*, well-known to be associated with proliferation, invasion, stemness, and resistance to apoptosis. (Li et al., 2024; Romanowicz and Łukaszewicz-Zając, 2025)

Crucially, experimental and epidemiological studies suggest that HPyVs can interfere with this molecular pathway (Zeng et al., 2020), particularly JCPyV. This virus has been reported in CRC tissues and implicated in oncogenic processes through interactions with pathways regulating cell-cycle progression and apoptosis. (Laghi et al., 1999; Hori et al., 2005; Lin et al., 2008; Link et al., 2009; Fang et al., 2022) Notably, JCPyV LTAg expression has been associated with dysregulation of β-catenin signaling, promoting its nuclear translocation and transcriptional activity in colorectal tumor cells. (Enam et al., 2002, 2006; Ripple et al., 2014) These findings support the hypothesis that JCPyVs-mediated modulation of the Wnt/β-catenin pathway may contribute to colorectal carcinogenesis. Consistently, evidence of SV40 and BKPyV infections has also been reported in CRC patients. (Campello et al., 2010; Dolci et al., 2021; Darwish et al., 2024) Further support for the involvement of HPyVs in CRC comes from a recent study that detected HPyV6 DNA, transcripts, and LT protein exclusively in the tumoral tissue of a patient with colon adenocarcinoma, reinforcing the clinical relevance of HPyVs in colorectal oncogenesis. (Dolci et al., 2026)

Among HPyVs, Merkel Cell Polyomavirus (MCPyV) is the only one with an established oncogenic role in human malignancies. MCPyV is a small non-enveloped virus, first discovered in 2008 (Feng et al., 2008), harbouring a double-stranded DNA genome composed of three functional regions: the early region, encoding the early proteins including LTAg, small T antigen (sTAg), 57 kT antigen, and an alternative LT open reading frame (ALTO), which are involved in viral replication and cellular transformation; the late region, encoding for the structural proteins Viral Protein (VP) 1 and VP2, and the viral microRNAs (miRNAs), miR-M1–5p and miR-M1–3p, potentially involved in the regulation of viral replication, and the non-coding control region (NCCR) containing the origin of viral replication and the regulatory elements. (Ahmed et al., 2021) MCPyV is highly prevalent in the global population, where it persists as a generally asymptomatic infection. During persistent infection, the virus replicates and exists as an episome within infected non-malignant cells. (Yang and You, 2022) Unlike other HPyVs whose oncogenic potential in humans remains debated, MCPyV is widely recognized as the primary causative agent of Merkel cell carcinoma (MCC), a highly aggressive cutaneous malignancy. (Houben et al., 2023) In MCC, clonally integrated viral DNA and the expression of a truncated form of the LTAg drive malignant transformation by disrupting key tumor suppressor pathways, such as the retinoblastoma (Rb) protein network. (Myrda et al., 2024) Given its potent transforming capabilities and its ability to establish persistent systemic infections, recent research has expanded beyond cutaneous oncology to investigate the presence and pathogenic contribution of MCPyV in non-MCC malignancies, including CRC. (Dimitraki and Sourvinos, 2022) Emerging evidence has identified MCPyV DNA within colorectal tumor specimens, although prevalence rates and clinical significance vary across studies. (Loyo et al., 2010; Campello et al., 2011; Dolci et al., 2021)

Notably, the molecular mechanisms through which MCPyV could contribute to colorectal tumorigenesis remain largely undefined. Considering the established role of β-catenin signaling in CRC and previous observations linking PyV infection to Wnt pathway dysregulation (Enam et al., 2002; Gan and Khalili, 2004; Ripple et al., 2014; Passerini et al., 2025), it is plausible that MCPyV may contribute to colorectal carcinogenesis through similar mechanisms.

Based on this background, the current study aimed to investigate the prevalence and the molecular profiling of MCPyV in CRC patients and to evaluate its possible role in Wnt/β-catenin modulation. Elucidating the relationship between MCPyV infection and molecular pathways involved in colorectal carcinogenesis may provide further insights into the potential contribution of viral factors to CRC biology and progression.

## 2 Materials and methods

### 2.1 Case study

The case study included 122 colorectal cancer patients who underwent surgery at four different hospitals: the Istituto Clinico Città Studi of Milano, Italy; the Habib Thameur Hospital, Tunis, Tunisia; ASST Valle Olona, Ospedale di Circolo, Busto Arsizio, Varese, Italy; and ASST Papa Giovanni XXIII Hospital, Bergamo, Italy. Patients’ demographic and clinical data were reported in Table 1. For each patient, normal peritumoral tissues (n = 122) and tumor tissues (n = 122) were analyzed. The study obtained approval from the Institutional Ethics Committees (Comitato Etico Istituto Clinico Città Studi, Ospedale Maggiore Policlinico, Milan, protocol number 683_2017bis; reg. 2023–0138, Ospedale Papa Giovanni XXIII, Bergamo) and was conducted in accordance with the WMA Declaration of Helsinki; all patients provided informed consent.

**Table 1:** Demographic and clinical characteristics of the enrolled patients.

**Variable**	**Population**
Patients	n = 122
Sex, n (%)^a^	
M	65
F	50
Age (years), median (IQR)^a^	73 (66.5–80)
Tumor stage*, n (%)^b^	
I	20
II	41
III	45
IV	6
Tumor location. n (%)^a^	
Right colon	81
Left colon	25
Rectum	6

^a^For 7 patients data were missing.

^b^For 10 patients data were missing.

*TNM classification.

### 2.2 DNA extraction

DNA was isolated from 20 mg of tumor and normal peri‑tumoral tissues using the QIAamp DNA Mini kit (Qiagen, Hilden, Germany), according to the manufacturer’s protocol.

### 2.3 Detection of MCPyV DNA by real-time and standard polymerase chain reaction

The presence of the MCPyV genome was analyzed using quantitative polymerase chain reaction (qPCR) on the 7500 Real-Time PCR System (Applied Biosystems, USA). The reaction was conducted in a final volume of 20 µL using the 2× Luna Universal Probe qPCR Master Mix (New England Biolabs, MA, USA), 200 nM of forward primer, 400 nM of reverse primer, 200 nM of probe, and 5 µL of DNA template. The amplification was performed with the following thermal cycling conditions: initial denaturation at 95 °C for 1 min, followed by 45 cycles of denaturation at 95 °C for 15 s and annealing at 60 °C for 30 s. The sequences of the primers and of the probe were the following: primer forward 5′-TGCCTCCCACATCTGCAAT-3′, reverse primer 5′-GTGTCTCTGCCAATGCTAAATGA-3′ and probe 5′-FAM-TGTCACAGGTAATATC-MGB-3′. Serial dilutions of standard controls and negative controls were included in each run. Viral copies were expressed as copies/ug. To calculate the percentage of the infected cells, qPCR targeting the β‐globin gene was performed as previously reported. (Bella et al., 2015) MCPyV-positive samples were further analyzed for selected viral regions, including NCCR, and VP1, by standard PCR. (Hashida et al., 2013; Passerini et al., 2024)

### 2.4 Sequence analysis of MCPyV NCCR, VP1, and LTAg

To detect possible rearrangements within viral regions, the amplified products of NCCR and VP1 were purified using the miPCR purification kit (Metabion, Planegg, Germany), sequenced (Bio-Fab research, Rome, Italy), and compared with the reference strain MCC350: EU375803 (GeneBank), using Clustal W2 (http://www.ebi.ac.uk/Tools/msa/clustalw2/). Moreover, viral regions corresponding to the entire LTAg were analyzed using six primer sets, followed by PCR product purification and sequencing. (Hashida et al., 2013)

### 2.5 Analysis of integration sites

The presence of integration sites was investigated employing the detection of the integrated papilloma sequence (DIPS)-PCR technique. (Sastre-Garau et al., 2009) Specifically, total DNA was digested with TaqI. DNA fragments were then ligated to enzyme-specific adaptors and subjected to PCR assays using viral- and adaptor-specific primers. (Hashida et al., 2013) Following purification and sequencing, the integration sites were assessed by comparing the obtained sequences with the databases of the National Center for Biotechnology Information (NCBI), using the Basic Local Alignment Search Tool (BLAST 2.17.0).

### 2.6 RNA extraction

RNA was isolated from 20 mg of tumor and adjacent healthy tissues using RNA Blood Mini Kit (Qiagen, Hilden, Germany), according to the manufacturer’s protocol.

### 2.7 Reverse-transcription and transcripts analysis

RNA was reverse-transcribed (RT) using the SensiFAST cDNA Synthesis kit (Meridian Bioscience, Cincinnati, OH, USA), and after *GAPDH* amplification for cDNA quality assessment, an aliquot of the RT mixture was used for PCR targeting the *LTAg* and *VP1* genes. (Hashida et al., 2013)

### 2.8 MCPyV miRNA detection

Viral miRNAs expression was evaluated using the pre-designed TaqMan microRNA assays (Thermo Fisher Scientific, Waltham, MA, USA) targeting mcv-miR-M1–5p (ID 006,356). In addition, the human RNU6B miRNA (ID001093) was included as an endogenous control.

### 2.9 Analysis of CTNNB1, MYC, CCND1 and AXIN2 genes

cDNA was further used to evaluate the expression of *CTNNB1, MYC, CCND1* and *AXIN2*. RT-qPCR was performed employing specific primers as previously described. (Sareddy et al., 2009; Knauthe et al., 2022; Passerini et al., 2025) To normalize mRNA expression levels, *GAPDH* was utilized as the housekeeping gene. Relative mRNA expression was calculated through the ΔCt method and expressed as 2^-ΔCt^.

### 2.10 Statistical analysis

For statistical analysis, data were reported as median and interquartile range (IQR). Viral loads and gene expression were assessed for normality using the Shapiro-Wilk test. Viral loads and relative levels of Wnt target genes were compared between groups using the Mann-Whitney U test. Categorical variables were reported as counts and percentages, and group comparisons were performed using the chi-square or Fisher's exact test, depending on the data distribution. Statistical significance was determined by a *p*-value *<*0.05.

## 3 Results

### 3.1 Detection of MCPyV DNA

MCPyV was isolated from 43/122 (35.2%) CRC patients (Table 2). Specifically, viral DNA was detected in 26 out of 122 (21.3%) tumor samples and 23 out of 122 (18.8%) adjacent healthy tissues, with median viral loads of 1.39×10^4^ (8.95×10^3^–6.31×10^4^) and 1.61×10^4^ (5.86×10^3^–2.25×10^4^) copies/µg, respectively. The positivity, the viral load, and the percentage of infected cells are reported in Table 2. No significant differences (*p* = 0.3) in viral load were observed when comparing virus-positive tumoral and healthy tissues (Table 2), or when restricting the analysis to the 6 patients positive in both matched tissues (*p* = 0.44). MCPyV-positivity and viral load were also evaluated in relation to clinical-pathological characteristics in both tumor and adjacent healthy tissues. No significant associations were observed between MCPyV positivity or viral load and age and tumor stage (Table 3). In contrast, MCPyV prevalence in tumor tissues was significantly correlated with anatomical site (*p* = 0.03), with a higher positivity rate observed in left-sided tumors compared to right-sided lesions (*p* = 0.024). Moreover, a significantly higher viral load was observed in tumor tissues from female patients (*p* = 0.036) and in low-grade tumors (*p* = 0.039).

**Table 2:** Summary of MCPyV prevalence, viral load and sequences in CRC patients.

	**Tumor tissue (n** **=** **122)**	**Peritumoral tissue (n** **=** **122)**	***p*-value**
**MCPyV Pos/Total (%)**	26/122 (21.3%)	23/122 (18.8%)	0.74
**MCPyV viral load (Median, IQR)**	1.39×10^4^ (8.95×10^3^–6.31×10^4^)	1.61×10^4^ (5.86×10^3^–2.25×10^4^)	0.3
**% infected cells (Median,IQR)**	4.6% (0.5%−20.3%)	4.0% (0.2%−12.7%)	0.55
**Canonical NCCR, n (%)**	26/26 (100%)	23/23 (100%)	-
**Canonical VP1, n (%)**	26/26 (100%)	23/23 (100%)	-
**Full-length LTAg, n (%)**	26/26 (100%)	23/23 (100%)	-

Viral load is expressed as copies/µg.

**Table 3:** Clinical pathological characteristics of MCPyV-positive tumor tissues and peritumoral tissues.

		**Peritumoral tissue**		**Tumor tissue**	
**Characteristics**	**Total**	**MCPyV+, n (%)**	**Viral Load (median, IQR)**	**MCPyV+, n (%)**	**Viral Load (median, IQR)**
Age (years)					
≤65	28	4 (14.3%)	1.62×10^4^ (5.4 × 10^3^–3.95×10^4^)	9 (32.1%)	1.29×10^4^ (5.3 × 10^3^–2.21×10^4^)
>65	87	18 (20.7%)	1.54×10^4^ (9.06×10^3^–6.9 × 10^4^)	17 (19.1%)	1.6 × 10^4^ (7.38×10^3^–2.8 × 10^4^)
p-value		0.49	0.43	0.17	0.37
Gender					
Male	65	14 (21.5%)	1.65×10^4^ (8.3 × 10^3^–8.3 × 10^4^)	16 (24.6%)	9.72×10^3^ (5.4 × 10^3^–1.88×10^4^)
Female	50	8 (16%)	1.5 × 10^4^ (6.1 × 10^3^–5.4 × 10^4^)	10 (20%)	2.46×10^4^ (1.18×10^4^–5.23×10^4^)
p-value		0.45	0.5	0.56	**0.036**
Anatomical Site					
Right Colon	81	13 (16%)	1.9 × 10^4^ (7.4 × 10^3^–10.9 × 10^5^)	13 (16%)	1.4 × 10^4^ (7 × 10^3^–4.07×10^4^)
Left Colon	25	6 (24%)	1.5 × 10^4^ (1.2 × 10^4^–5 × 10^4^)	10 (40%)	8.16×10^3^ (5.76×10^3^–1.75×10^4^)
Rectum	9	3 (33.3%)	8.95×10^3^ (4.8 × 10^3^–4.4 × 10^4^)	3 (33.3%)	2.16×10^4^ (1.29×10^4^–3.27×10^4^)
p-value		0.27	0.49	**0.03**	0.34
Tumor Stage					
Stage I / II	61	12 (19.7%)	1.65×10^4^ (6.2 × 10^3^–4.5 × 10^4^)	13 (21.3%)	1.94×10^4^ (1.1 × 10^4^–2.8 × 10^4^)
Stage III / IV	51	9 (17.6%)	1.5 × 10^4^ (9.96×10^3^–1.1 × 10^5^)	13 (25.5%)	8.74×10^3^ (5.2 × 10^3^–1.65×10^4^)
p-value		0.78	0.6	0.6	**0.039**

IQR: interquartile range.

### 3.2 Sequencing and integration sites analysis

Across all the MCPyV-positive samples, the amplified NCCR and VP1 regions displayed canonical sequences, showing 100% nucleotide identity with the reference strain deposited in GenBank MCC350 (EU375803) (Table 2). Moreover, the full-length form of *LTAg* was detected with no evidence of viral integration.

### 3.3 Detection of viral transcripts

When evaluating MCPyV-positive samples for *LTAg* and *VP1* transcripts, both genes were expressed in all specimens. Among virus-positive samples, mcv-miR-M1–5p was detected only in one (1/26, 3.8%) tumoral tissue (Supplementary Table 1).

### 3.4 Relative expression of CTNNB1, MYC, CCND1 and AXIN2 genes

To investigate the Wnt/β-catenin pathway in colorectal tissues, the mRNA expression levels of key factors, including *CTNNB1, MYC, CCND1* and *AXIN2* were evaluated. All four genes were significantly over-expressed in MCPyV-positive tissues compared to negative ones in both peritumoral and tumoral compartments (Fig. 1; Panel A, *p* < 0.05). In addition, to determine whether this virus-mediated upregulation was restricted to specific clinical subcategories, tumor specimens were stratified according to anatomical site and tumor stage. mRNA levels of *CTNNB1, MYC, CCND1* and *AXIN2* remained significantly higher in MCPyV-positive tissues across all tumor locations (Fig. 1; Panel B, *p* < 0.05). Similarly, higher transcript levels of these cellular genes were reported in virus-positive tissues, compared to negative ones, regardless of tumor stage (Fig. 1; Panel C, *p* < 0.05). Furthermore, among MCPyV-positive tissues, no statistically significant differences were observed based on either anatomical site or tumor stage (*p* > 0.05).

**Fig. 1:**
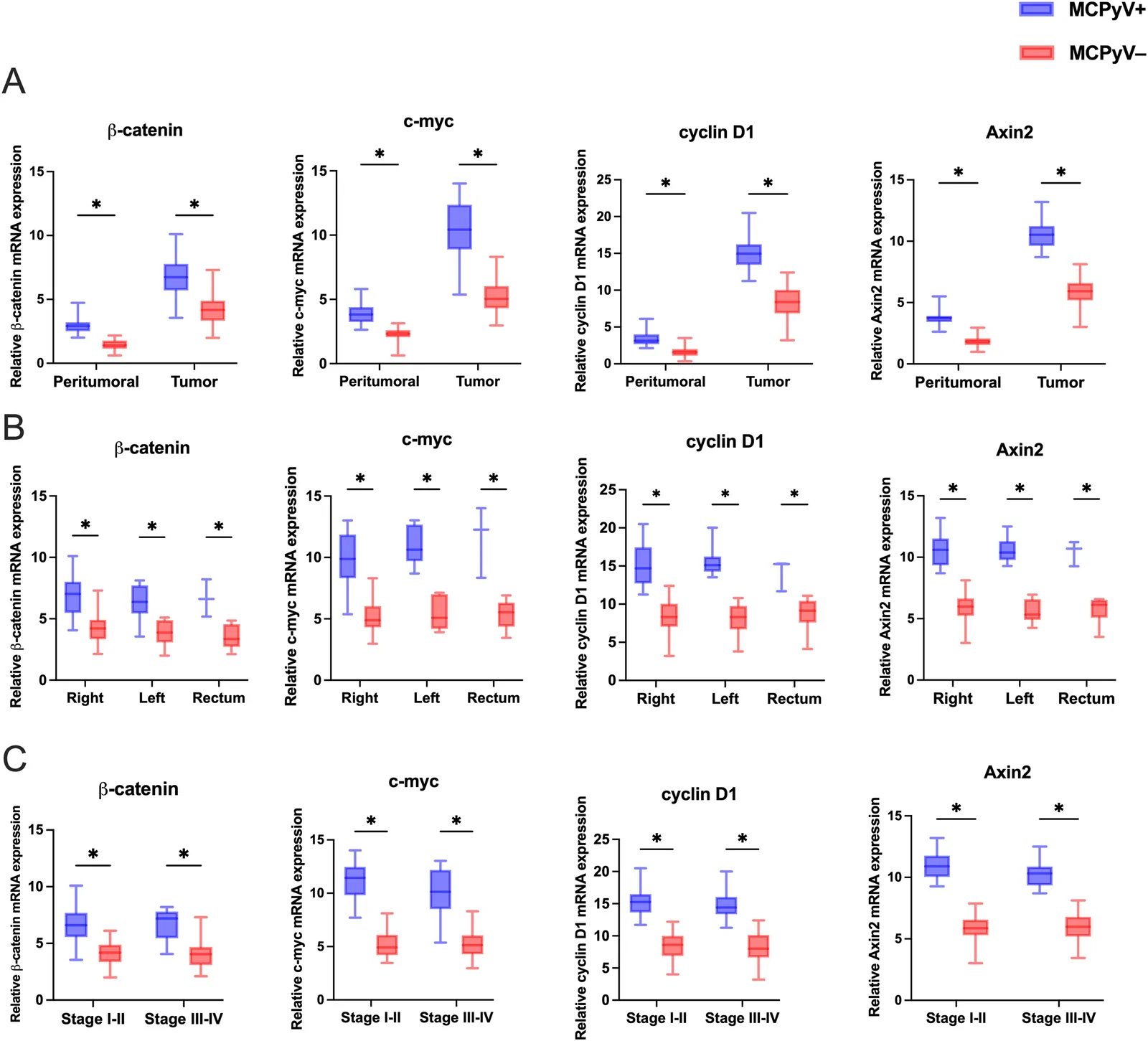
Relative expression of *β-catenin (CTNNB1), c-myc (MYC), cyclin D1* (*CCND1*) and *Axin2 (AXIN2)* genes in MCPyV-positive and negative tissues. (**A**) Comparison between peritumoral tissues, (**B**) Stratification of tumor tissues based on anatomical site, (**C**) Stratification of tumor tissues based on tumor stage. Data are expressed as box-and-whiskers plots, representing the median and the interquartile range (IQR). Statistical significance was defined using the Mann-Whitney test. *p < 0.05.

## 4 Discussion

CRC is a widespread malignancy driven by several risk factors. Among them, microbial infections play a major role by altering the intestinal microenvironment, thus contributing to chronic inflammation and malignant transformation. (Modeel et al., 2025) In this framework, oncogenic viruses have gained increasing recognition for their role in CRC carcinogenesis, particularly due to their ability to disrupt the cell cycle. (Marongiu and Allgayer, 2022) Among them, MCPyV is well known to be associated with the aggressive skin cancer MCC, although a putative role in other malignancies has also been proposed. (Dimitraki and Sourvinos, 2022) Like other HPyVs, MCPyV has been detected in the gastrointestinal tract, suggesting a plausible oro-fecal transmission route. (Loyo et al., 2010; Prezioso et al., 2019) Furthermore, despite the isolation of MCPyV from CRC tissues (Campello et al., 2011; Dolci et al., 2021), current literature remains limited to viral DNA detection. Consequently, there is a critical need for comprehensive molecular characterization to examine whether MCPyV actively replicates or drives oncogenesis in these tissues.

To address this, here we investigated the presence and the molecular profiling of MCPyV in a cohort of 122 patients with a diagnosis of CRC. Viral DNA was detected in approximately 35% patients with a similar distribution between tumor (21.3%) and peritumoral tissues (18.8%). Notably, viral load did not differ between cancerous and adjacent healthy tissues, suggesting that MCPyV establishes a widespread infection throughout the colorectal mucosa, which may serve as a reservoir for viral persistence within the gastrointestinal tract. Although MCPyV prevalence displayed no significant correlation with patient age or tumor stage, distinct associations with clinical-pathological features emerged. Viral distribution was significantly correlated with tumor site, being enriched in left-sided tumors. Since proximal and distal CRCs arise from distinct embryological origins and retain different molecular profiles (Missiaglia et al., 2014), our findings suggest that the distal colon may provide a more permissive environment for MCPyV infection. Moreover, significantly higher viral loads were observed in low-grade tumors, indicating that a more stable cellular environment may favor MCPyV replication than in high-grade, poorly differentiated malignancies.

Once the viral prevalence was determined, sequencing of key genomic regions was performed to characterize MCPyV infection in colorectal tissues better, revealing canonical NCCR and VP1 structures. In particular, we found no evidence of clonal integration and LTAg truncation, which are well-defined hallmarks of MCPyV-positive MCCs. (Feng et al., 2008) Moreover, transcriptional profiling demonstrated the expression of both early (*LTAg*) and late (*VP1*) genes, whereas MCPyV-encoded miRNAs were detected in only one tumor tissue. In MCC, tumorigenesis is driven by integrated, replication-defective viral genomes that retain transforming properties. (Spurgeon and Lambert, 2013; Ahmed et al., 2021) Conversely, the detection of early and late transcripts, with no signs of viral integration in our cohort, is consistent with previous data from non-MCC malignancies, where MCPyV maintains an episomal and transcriptionally active state. (Dimitraki and Sourvinos, 2022; Passerini et al., 2024) These molecular features indicate that MCPyV acts as a chronic, transcriptionally active resident of the colorectal mucosa, rather than a transient bystander acquired through the oro-fecal route. (Campello et al., 2011)

To explore whether this persistent state is involved in oncogenic processes, a further objective of this study was to investigate the Wnt/β-catenin pathway. Aberrant activation of the Wnt signaling pathway is recognized as a key driver of colorectal carcinogenesis, typically caused by loss of function of the APC tumor suppressor gene. (Li et al., 2024; Zhao et al., 2022) Our data showed significant overexpression of *CTNNB1* and its key downstream oncogenic targets, *MYC* and *CCND1,* in virus-positive tissues, suggesting a plausible role for MCPyV in perturbing the transcriptional output of this critical signaling cascade and thus potentially modulating host signaling pathways relevant to colorectal biology. Whether MCPyV plays any etiological role in colorectal carcinogenesis, or whether its detection is coincidental, remains to be established by future prospective case-control studies. This virus-associated up-regulation was maintained across tumor and adjacent normal tissues. Furthermore, stratification by anatomical site and tumor stage revealed consistent overexpression of Wnt target genes across all virus-positive samples, with no statistically significant differences among the clinical subgroups.

As assessment of nuclear β-catenin staining was not feasible, *AXIN2* expression was used as a surrogate marker to further evaluate pathway activation. (Rogers et al., 2013) The obtained results revealed a pattern comparable with the previous examined genes, showing a higher expression in MCPyV-positive tissues.

Taken together, these findings strongly support the role of MCPyV in manipulating the Wnt signaling cascade rather than representing a bystander in colorectal tissues. Mechanistically, the enhanced expression of *CTNNB1* and its downstream effectors may reflect a model involving cytoplasmic stabilization and subsequent nuclear translocation of *CTNNB1,* which in turn stimulates transcription of *MYC, CCND1* and *AXIN2*.

Given the transcriptional activity of the viral genomes and the possibility of their persistence in an episomal form, it is plausible that wild-type MCPyV oncoproteins interfere with the host disruption complex or directly stabilize β-catenin, as previously demonstrated for JCPyV. (Moens and Macdonald, 2019) Specifically, the sT protein may bind and inhibit the protein phosphatase 2A (PP2A), a critical regulator of the β-catenin destruction complex, thereby preventing its phosphorylation and subsequent proteasomal degradation. (Thompson and Williams, 2018) Concurrently, full-length LT could interact with cytoplasmic β-catenin, promoting its translocation to the nucleus, where it functions as a transcriptional co-activator, as described for other human viruses. (Marongiu and Allgayer, 2022)

Indeed, this molecular hijacking of the Wnt/β-catenin pathway represents a conserved strategy among oncogenic viruses. EBV stabilizes β-catenin via LMP1 by reducing its proteasomal degradation (Shackelford et al., 2003; Jang et al., 2005) and high-risk HPVs activate Wnt/β-catenin signaling through E6 and E7 oncoproteins by interfering with β-catenin degradation and enhancing β-catenin/TCF-dependent transcription (Bello et al., 2015) Within the *Polyomaviridae* family, JCPyV has been implicated in colorectal oncogenesis through LT-mediated deregulation of Wnt signaling (Enam et al., 2002; Ripple et al., 2014), whereas SV40 perturb this pathway by interacting with PP2A. (Sablina et al., 2010) Our findings extend a similar mechanism to that of MCPyV, showing overexpression of Wnt target genes associated with viral infection. Interestingly, an analogous molecular signature was observed in JCPyV-positive brain tumors (Passerini et al., 2025), indicating a conserved HPyVs-driven mechanism in altering the Wnt/β-catenin signaling across different malignancies. Overall, these data suggest that persistent MCPyV infection could contribute to sustained modulation of the Wnt/β-catenin cascade. This chronic signaling deregulation may promote mucosal proliferation and support a possible role for MCPyV as a cofactor in colorectal carcinogenesis.

## 5 Conclusions and limitations

In conclusion, our findings indicate the presence of MCPyV in colorectal tissues and support a plausible role for this virus in the colorectal tumor microenvironment. By modulating Wnt/β-catenin signaling at the transcriptional level, MCPyV may contribute to the establishment of a pro-proliferative phenotype.

Although these results provide new insights into the role of MCPyV in the colorectal oncogenic landscape, some limitations should be acknowledged. First, limited tissue availability prevented the cloning and sequencing of full-length MCPyV genomes. Although the NCCR, VP1, and full-length LTAg regions were detected without evidence of integration or truncating mutations, these findings do not establish the presence of intact episomal genomes, which remains a hypothesis requiring confirmation by full-length viral genome characterization. Second, the low proportion of MCPyV-infected cells raises the possibility that the observed transcriptional differences may reflect regional tissue characteristics or differences in cellular composition rather than a direct effect of viral infection. Third, the absence of a CRC-free control group prevents to determine whether MCPyV prevalence is specifically enriched in CRC patients compared with the general population. Finally, RT-qPCR offers information on mRNA expression, but additional proteomic analyses and immunohistochemical studies will be required to detect LTAg protein and assess β-catenin expression and nuclear localization. The lack of LTAg protein-level validation limits our ability to determine whether MCPyV is biologically active in the analyzed tissues, as detection of viral transcripts alone does not demonstrate the production of viral proteins. Similarly, although the observed transcriptional changes are consistent with Wnt pathway activation, they do not confirm signaling activation. Therefore, the present findings should be interpreted as evidence of transcriptional dysregulation rather than definitive proof of active viral replication or functional Wnt pathway activation.

For this reason, future studies will focus on more comprehensive characterization of the MCPyV genome and proteins and protein-level validation of Wnt signaling in virus-positive tumors. In parallel, the development of cellular models will be essential to dissect the molecular links between MCPyV infection and Wnt/β-catenin signaling.

## Author statement

We declare that this manuscript is original, has not been published before and is not currently being considered for publication elsewhere. We confirm that the manuscript has been read and approved by all named authors and that there are no other persons who satisfied the criteria for authorship but are not listed. We further confirm that all have approved the order of authors listed in our manuscript. We understand that the Corresponding Authors are the sole points of contact for the Editorial process. They are responsible for communicating with the other authors about progress, submissions of revisions, and final approval of proofs.

## Research funding

This research was supported by funds from the Italian Ministry of University and Research (MIUR) Research Grant (RP124190255A2CB7).

## Ethics statement

The study obtained approval from the Institutional Ethics Committees (Comitato Etico Istituto Clinico Città Studi, Ospedale Maggiore Policlinico, Milan, protocol number 683_2017bis; reg. 2023–0138, Ospedale Papa Giovanni XXIII, Bergamo) and was conducted in accordance with the WMA Declaration of Helsinki.

## Consent

All participants provided written informed consent.

## Ethics declaration

### Informed consent and patient details

Written informed consent to take part in the study and to publish the article has been obtained from all participants or their legal representatives. The privacy rights of participants have been observed.

### Human and biological material

This study included organ or tissue donors. This study includes human biological material and consent was obtained by donors, or their next of kin or legal representatives, for use in this study and for publication of the article. The samples used in this research were not sourced from executed prisoners or prisoners of conscience.

### Studies in human

This study was performed in compliance with relevant laws, regulatory frameworks and guidelines where the research took place. This study was approved by the Comitato Etico Istituto Clinico Città Studi, Ospedale Maggiore Policlinico; Ospedale Papa Giovanni XXIII, Bergamo. (Approval No 683_2017bis; reg. 2023–0138)

## CRediT authorship contribution statement

**Sara Passerini:** Writing – review & editing, Writing – original draft, Supervision, Investigation, Data curation, Conceptualization. **Maria Dolci:** Writing – review & editing, Investigation, Data curation. **Sara Messina:** Investigation, Data curation. **Valentina Alyssa Caterina:** Investigation, Data curation. **Lucia Signorini:** Investigation, Data curation. **Francesca Camurri:** Investigation, Data curation. **Marco Graziani:** Investigation, Data curation. **Giorgia Gallo:** Investigation, Data curation. **Giovanni Di Nardo:** Writing – review & editing, Supervision. **Serena Delbue:** Writing – review & editing, Supervision, Conceptualization. **Valeria Pietropaolo:** Writing – review & editing, Writing – original draft, Supervision, Funding acquisition, Conceptualization.

## Declaration of competing interest

The authors declare that they have no known competing financial interests or personal relationships that could have appeared to influence the work reported in this paper.

## Data Availability

Data will be made available on request.
