# Supplementary material for: Merkel Cell Polyomavirus in colorectal cancer: exploring the Wnt/β-catenin pathway
Source: Virus Res. 2026 Sep 24;373:199809. doi: 10.1016/j.virusres.2026.199809 (PMC13639745; doi:10.1016/j.virusres.2026.199809)
Supplement: Supplementary file 1 [file mmc1.docx]

**Supplementary Table 1.**

Summary of Merkel Cell Polyomavirus (MCPyV) prevalence, viral load and transcriptional profiling in virus-positive specimens.

| **Patient ID** | **Age** | **Sex** | **Tumor stage** | **Tumor site** | **Peritumoral tissues** | | | | | | **Tumor tissues** | | | |  |  |
| --- | --- | --- | --- | --- | --- | --- | --- | --- | --- | --- | --- | --- | --- | --- | --- | --- |
|  |  |  |  |  | **MCPyV** | **Viral load**  **(copies/µg)** | **% infected cells** | **Viral transcripts** | | | **MCPyV** | **Viral load**  **(copies/µg)** | **% infected cells** | **Viral transcripts** | | |
|  |  |  |  |  |  |  |  | **LTAg** | **VP1** | **miR-5p** |  |  |  | **LTAg** | **VP1** | **miR-5p** |
| ICCS#14 | 69 | M | III | left | + | 1.41×10^4^ | 2.25×10^-3^ | + | + | ‒ | + | 1.69×10^4^ | 6.78×10^-5^ | + | + | ‒ |
| ICCS#22 | 85 | M | II | left | + | 1.44×10^4^ | 3.02×10^-2^ | + | + | ‒ | ‒ |  |  |  |  |  |
| ICCS#35 | 88 | F | III | right | + | 1.46×10^4^ | 3.4×10^-4^ | + | + | ‒ | ‒ |  |  |  |  |  |
| ICCS#41 | 84 | M | III | left | ‒ |  |  |  |  |  | + | 6.02×10^3^ | 1.17×10^-2^ | + | + | ‒ |
| ICCS#42 | 66 | M | III | right | ‒ |  |  |  |  |  | + | 3.68×10^3^ | 6.8×10^-3^ | + | + | ‒ |
| ICCS#43 | 87 | M | III | right | + | 1.38×10^5^ | 1.6×10^-1^ | + | + | ‒ | + | 8.74×10^3^ | 1×10^-1^ | + | + | ‒ |
| ICCS#80 | 74 | M | II | right | + | 1.40×10^5^ | 8.39×10^-2^ | + | + | ‒ | ‒ |  |  |  |  |  |
| # 18243 | NA | NA | NA |  | + | 1.74×10^4^ | 4.7×10^-2^ | + | + | ‒ | ‒ |  |  |  |  |  |
| #16-I-60 | 65 | F | I | right | + | 2.67×10^4^ | 9.96×10^-2^ | + | + | ‒ | ‒ |  |  |  |  |  |
| #514-17 | 85 | F | II | right | ‒ |  |  |  |  |  | + | 1.65×10^4^ | 3.93×10^-1^ | + | + | + |
| #13701/16 | 91 | F | II | right | ‒ |  |  |  |  |  | + | 6.62×10^4^ | 3.21×10^0^ | + | + | ‒ |
| P36 | 64 | M | III | left | ‒ |  |  |  |  |  | + | 5.12×10^3^ | 4.64×10^-3^ | + | + | ‒ |
| P65 | 59 | M | II | rectum | + | 4.37×10^4^ | 4.39×10^-2^ | + | + | ‒ | ‒ |  |  |  |  |  |
| P83 | 51 | F | III | rectum | ‒ |  |  |  |  |  | + | 3.27×10^4^ | 3.22×10^-2^ | + | + | ‒ |
| BG 1 | 65 | M | II | left | ‒ |  |  |  |  |  | + | 2.26×10^4^ | 2.01×10^-1^ | + | + | ‒ |
| BG 3 | 58 | F | III | right | ‒ |  |  |  |  |  | + | 1.38×10^4^ | 1.11×10^-1^ | + | + | ‒ |
| BG 5 | 67 | M | III | right | + | 1.43×10^4^ | 4.16×10^-2^ | + | + | ‒ | ‒ |  |  |  |  |  |
| BG 15 | 87 | F | III | right | + | 8.05×10^4^ | 1.94×10^0^ | + | + | ‒ | ‒ |  |  |  |  |  |
| BG 18 | 75 | M | II | left | ‒ |  |  |  |  |  | + | 1.94×10^4^ | 6.59×10^-2^ | + | + | ‒ |
| BG 21 | 77 | M | IV | right | + | 1.83×10^4^ | 6.41×10^-1^ | + | + | ‒ | ‒ |  |  |  |  |  |
| BG 22 | 51 | M | I | rectum | ‒ |  |  |  |  |  | + | 2.16×10^4^ | 2.61×10^-1^ | + | + | ‒ |
| BG 23 | 93 | F | II | right | + | 6.31×10^4^ | 3.41×10^-1^ | + | + | ‒ | + | 6.51×10^4^ | 9.36×10^-1^ | + | + | ‒ |
| BG 25 | 68 | M | NA | left | + | 6.41×10^4^ | 3.08×10^-1^ | + | + | ‒ | ‒ |  |  |  |  |  |
| BG34 | 69 | F | III | right | ‒ |  |  |  |  |  | + | 4.80×10^4^ | 2.11×10^-1^ | + | + | ‒ |
| BG36 | 75 | M | II | left | + | 4.52×10^4^ | 9.4×10^-2^ | + | + | ‒ | ‒ |  |  |  |  |  |
| BG37 | 80 | M | III | left | ‒ |  |  |  |  |  | + | 1.60×10^4^ | 8.19×10^-2^ | + | + | ‒ |
| BG38 | 76 | M | II | right | ‒ |  |  |  |  |  | + | 2.25×10^4^ | 6.32×10^-2^ | + | + | ‒ |
| BG42 | 74 | M | II | right | + | 1.86×10^4^ | 1.27×10^-1^ | + | + | ‒ | ‒ |  |  |  |  |  |
| BG52 | 73 | F | II | rectum | + | 8.95×10^3^ | 3.57×10^-2^ | + | + | ‒ | ‒ |  |  |  |  |  |
| BG54 | 74 | F | II | right | ‒ |  |  |  |  |  | + | 3.34×10^4^ | 3.18×10^-1^ | + | + | ‒ |
| BG62 | 59 | M | II | rectum | ‒ |  |  |  |  |  | + | 1.29×10^4^ | 1.03×10^-1^ | + | + | ‒ |
| BG70 | 86 | F | III | left | + | 1.61×10^4^ | 3.62×10^-3^ | + | + | ‒ | ‒ |  |  |  |  |  |
| BG74 | 62 | M | III | right | + | 5.75×10^3^ | 6.29×10^-4^ | + | + | ‒ | ‒ |  |  |  |  |  |
| BG76 | 70 | M | III | left | + | 5.81×10^3^ | 4.97×10^-4^ | + | + | ‒ | + | 1.03×10^4^ | 3.94×10^-3^ | + | + | ‒ |
| BG81 | 71 | M | III | left | ‒ |  |  |  |  |  | + | 5.84×10^3^ | 2.55×10^-3^ | + | + | ‒ |
| BG91 | 87 | F | II | rectum | + | 4.81×10^3^ | 2×10^-3^ | + | + | ‒ | ‒ |  |  |  |  |  |
| BG94 | 87 | M | IV | right | ‒ |  |  |  |  |  | + | 5.25×10^3^ | 1.62×10^-3^ | + | + | ‒ |
| BG96 | 58 | M | III | right | ‒ |  |  |  |  |  | + | 5.13×10^3^ | 1.92×10^-3^ | + | + | ‒ |
| BG97 | 45 | F | II | left | ‒ |  |  |  |  |  | + | 5.50×10^3^ | 1.74×10^-3^ | + | + | ‒ |
| BG102 | 55 | M | I | right | + | 5.29×10^3^ | 4.45×10^-3^ | + | + | ‒ | ‒ |  |  |  |  |  |
| BG105 | 71 | M | I | right | + | 9.10×10^3^ | 1.95×10^-3^ | + | + | ‒ | + | 9.13×10^3^ | 9.17×10^-3^ | + | + | ‒ |
| BG107 | 46 | F | II | left | ‒ |  |  |  |  |  | + | 5.87×10^3^ | 5.06×10^-3^ | + | + | ‒ |
| BG113 | 90 | F | II | right | + | 5.19×10^3^ | 1.44×10^-3^ | + | + | ‒ | + | 1.40×10^4^ | 1.91×10^-2^ | + | + | ‒ |

NA: not available
